# Synthetic Antiferromagnetic Designer Nanodisks for High‐Performance Magnetic Separation

**DOI:** 10.1002/adhm.202500616

**Published:** 2025-08-11

**Authors:** Subas Scheibler, Sebastian Habermann, Alexander Gogos, Santiago Helbig, Lukas R. H. Gerken, Anna L. Neuer, Vera M. Kissling, Michal Krupinski, Mohammad Alinezhadfar, Rowena Crockett, Nico Kummer, Erik M. Mayr, Dieter Süss, Hans J. Hug, Inge K. Herrmann

**Affiliations:** ^1^ Nanoparticle Systems Engineering Laboratory, Institute of Energy and Process Engineering (IEPE) Department of Mechanical and Process Engineering (D‐MAVT), ETH Zurich Sonneggstrasse 3 Zurich 8092 Switzerland; ^2^ Nanomaterials in Health Laboratory, Department of Materials Meet Life Swiss Federal Laboratories for Materials Science and Technology (Empa) Lerchenfeldstrasse 5 St. Gallen 9014 Switzerland; ^3^ Magnetic & Functional Thin Films Laboratory, Department of Materials Meet Life Swiss Federal Laboratories for Materials Science and Technology (Empa) Dübendorf 8600 Switzerland; ^4^ Faculty of Medicine University of Zurich Raemistrasse 71 Zurich 8006 Switzerland; ^5^ Ingenuity Lab Balgrist University Hospital Forchstrasse 340 Zurich 8008 Switzerland; ^6^ Physics of Functional Materials, Faculty of Physics University of Vienna Boltzmanngasse 5 Vienna 1090 Austria; ^7^ Research Platform MMM Mathematics – Magnetism – Materials University of Vienna Oskar‐Morgenstern‐Platz 1 Vienna 1090 Austria; ^8^ Institute of Nuclear Physics Polish Academy of Sciences Department of Magnetic Materials and Nanostructures ul. Radzikowskiego 152 Krakow 31‐342 Poland; ^9^ Surface Science and Coating Technologies Laboratory, Department of Advanced Materials and Surfaces Swiss Federal Laboratories for Materials Science and Technology (Empa) Dübendorf 8600 Switzerland; ^10^ Durability of Engineering Materials, Institute for Building Materials (IfB), Department of Civil, Environmental and Geomatic Engineering (D‐BAUG) ETH Zurich Zurich 8093 Switzerland; ^11^ Laboratory of Food Process Engineering, Institute of Food, Nutrition and Health (IFNH) Department for Health Sciences and Technology (D‐HEST) ETH Zurich Schmelzbergstrasse 9 Zurich 8092 Switzerland; ^12^ Laboratory for Cellulose and Wood Materials, Department of Materials Meet Life Swiss Federal Laboratories for Materials Science and Technology (Empa) Überlandstrasse 129 Dübendorf 8600 Switzerland; ^13^ Department of Physics University of Basel Basel CH‐4056 Switzerland

**Keywords:** disk‐shaped, magnetic capturing, synthetic antiferromagnetic particles, thin magnetic films

## Abstract

Magnetic separation of magnetic particles offers an appealing route to rapid and selective target capturing and isolation. However, to leverage such an approach to its full potential, high colloidal stability of the nanoparticles in the absence of a magnetic field for optimal binding, and rapid and quantitative recovery upon magnetic gradient field application are imperative. While these properties are mutually exclusive for conventional nanoparticles synthesized by wet‐chemistry approaches, sputter deposition gives access to layered architectures featuring the properties of a synthetic antiferromagnet (SAF, no magnetization in zero field, high magnetization upon field application).

Here, micromagnetic‐modelling based design optimization and scalable manufacturing of metallic CoSm‐based, metal oxide‐capped SAF magnetic disk particles (SAF MDPs) with high chemical and colloidal stability and cytocompatibility are presented. It is demonstrated that the SAF MDPs can be rapidly and much more efficiently separated (>99%) from flowing fluids (approx. 1 mL/min) compared to corresponding gold standard iron oxide beads (60% recovery), paving the way to quantitative capturing and enrichment of target compounds in high‐throughput conditions compliant with clinical and industrial applications.

## Graphical abstract

Micromagnetic‐modeling‐based design and scalable fabrication of synthetic antiferromagnet (SAF) magnetic disk particles (MDPs) using sputter deposition enable high‐performance magnetic separation. The SAF MDPs achieve high colloidal stability and magnetic responsiveness and enable efficient magnetic separation, outperforming conventional particles.

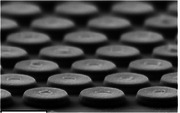

## 1 Introduction

Magnetic micro‐ and nanoparticles have enabled fascinating applications in biomedicine.^[^ ^1^ ^]^ Amongst them, magnetic separation is a promising technology enabling rapid and specific capturing and enrichment of target components (small molecules, proteins, bacteria, cells) from complex fluids, including blood.^[^ ^2^, ^3^, ^4^, ^5^, ^6^ ^]^ In biomedical diagnostics, sample purification is an essential step in which the specific target is extracted from a complex biological sample containing various components to then allow further analysis of the target compound in low‐matrix conditions. Magnetic isolation of the target compound by magnetic capturing, offers high throughput at low cost, is energy saving^[^ ^7^ ^]^ (minimal reliance on energy‐consuming equipment when permanent magnets are used) and offers uniquely high specificity, selectivity and efficiency. Magnetic separation has enabled fascinating applications, including the in vitro analysis of circulating tumour cells for diagnostic purposes (e.g., CellSearchTM,^[^ ^8^ ^]^ and Parithera^[^ ^9^ ^]^) and more recently, extracorporeal magnetic blood purification for the treatment of sepsis (hemotune AG^[^ ^10^ ^]^ and MediSieve Limited^[^ ^11^ ^]^). Particularly the capturing and subsequent detection and identification of pathogens with low abundancy in urine or blood, and subsequent magnetic upconcentration (enrichment) prior to detection is highly appealing, as time to pathogen identification (diagnosis), and early initialization of the adequate antibiotic therapy has critical impact on patient outcome and reduction of antimicrobial resistance (AMR).^[^ ^5^, ^12^, ^13^ ^]^ Despite encouraging achievements by both the research community and industry, the efficient magnetic separation of the magnetic nanoparticles remains a major concern in all of the aforementioned applications. The currently used particles are typically chemically synthesized, superparamagnetic iron oxide nanoparticles.^[^ ^14^ ^]^ Due to their superparamagnetic properties, these particles have a vanishing magnetic moment in zero magnetic field and thus exhibit a colloidal stability beneficial for the capturing of target compounds. However, because of the low magnetization of iron oxide and their superparamagnetic properties with magnetic susceptibility strongly affected by the size distribution arising from the chemical synthesis, the particles only develop a low magnetic moment in an applied magnetic field.^[^ ^15^ ^]^ Moreover, the magnetic moment grows with the particle volume, while its Stokes friction in liquid depends on its cross‐sectional area. Larger particle volumes (filled with magnetic material) would thus be beneficial. However, above a critical size, the magnetization of the particles becomes locked along their anisotropy axis, superparamagnetic properties are lost, and the particles become ferromagnetic, leading to destabilization of the particle suspension. The maximum volume and with it the achievable maximum moment of the particles thus remains limited. Consequently, the force acting on the particles in a magnetic gradient field is limited, and the particles can only be separated poorly from suspensions.

To overcome these limitations, researchers have developed supraparticles consisting of thousands of encapsulated or chemically cross‐linked networks of small, superparamagnetic metal oxide nanoparticles.^[^ ^16^, ^17^, ^18^ ^]^ However, such supraparticles are limited in packing density of the magnetic particles, and hence the magnetic volume per particle volume is typically low, leading to saturation magnetizations in the order of a few ten to hundreds kA/m (theoretical maximum 480 kA/m corresponding to the bulk saturation magnetization of magnetite^[^ ^15^ ^]^). While larger particles are highly beneficial from a magnetic separation point of view, there is a trade‐off between magnetic separability and surface area available for target immobilization. Thus, instead of creating supraparticles with unfavorably low specific surface area, ferromagnetic iron nanoparticles with ultra‐high saturation magnetization have been developed, which can be captured at impressively high efficiencies and speeds,^[^ ^19^ ^]^ however, face severe agglomeration issues, even in absence of a magnetic field, due to magnetic dipole–dipole interactions between particles.

The design and development of magnetic particles with optimized properties and geometries, such as thin disks with a maximized surface‐to‐volume ratio, is highly attractive. Especially top‐down fabricated, vortex or synthetic antiferromagnetic disk nanoparticles (SAF MDPs) offer a large volume (compared to small superparamagnetic particles), obtain a high magnetic moment in a sufficiently strong applied magnetic field, enabling an efficient magnetic separation, and have vanishing moment at zero field for a good magnetic particle suspension stability.

Here, we investigate SAF MDP with in‐plane magnetization for filtering applications. SAF MDPs have already been fabricated by various groups,^[^ ^20^ ^]^ e.g., for biomedical applictions.^[^ ^21^, ^22^ ^]^ Two types of SAF MDP systems have been realized, either with the magnetization in the particle plane^[^ ^23^, ^24^, ^25^, ^26^ ^]^ or along the perpendicular axis.^[^ ^27^, ^28^, ^29^, ^30^ ^]^ The latter is achieved by multilayers generating a perpendicular magnetic interfacial anisotropy, for example, occurring at the Co/Pt‐interface. While large magnetic torques were beneficial for a magneto‐mechanical cancer‐cell destruction^[^ ^28^, ^31^ ^]^ and can be achieved with the latter SAF MDPs, these contain a large volume fraction of expensive non‐magnetic materials. These costly Ru or Pt interlayers make such SAF designs economically less attractive for particle‐based filtering applications. In contrast, SAF MDP with in‐plane magnetization can be designed based on high contents of magnetic material and in‐expensive layers separating the two antiferromagnetically coupled magnetic layers. However, to date, no true turn‐on/turn‐off magnetization versus field behavior (sharp switching) has yet been reported for SAF MDP with in‐plane magnetization. Instead, linear and hysteresis‐free magnetization loops have typically been observed.

In our work, we leverage micromagnetic simulations to optimize the relevant system parameters for in‐plane magnetization SAF MDP to obtain a turn‐on/turn‐off behavior in a 500 nm‐diameter SAF MDP containing two high magnetization ferromagnetic layers. To fabricate such SAF MDPs, we present a nanosphere lithography approach using water‐air interface self‐assembly of polystyrene beads,^[^ ^32^ ^]^ offering a route towards more affordable production of high‐quality MDPs with narrow size distribution.^[^ ^33^ ^]^ We demonstrate that encapsulation of the SAF MDPs is instrumental in avoiding metal leaching (resulting in magnetic property loss) and at the same time offers a platform for straightforward surface functionalization. The freedom of choice in capping materials for SAF MDP gives large flexibility in surface chemistry and opens the door to lateral transfer of functionalization methods readily established for the functionalization of metal and metal oxide particles (especially silane chemistry). Finally, we present stable SAF MDPs optimized for magnetic separation, which are well tolerated by human cells and can be readily functionalized, enabling high‐performance magnetic capturing and separation.

## 2 Results and Discussion

### 2.1 Micromagnetic Simulations

The micromagnetic state of small disk‐shaped magnetic particles depends on the disk diameter, its thickness and the magnetic properties of the magnetic layer, such as the magnetization, the exchange stiffness and the magnetic anisotropy. Generally, small diameter particles with sufficiently small thicknesses develop a uniform monodomain state,^[^ ^34^ ^]^ whereas a multi‐domain or vortex state develops for larger diameter particles and thicknesses.^[^ ^35^, ^36^ ^]^

**Figure 1:**
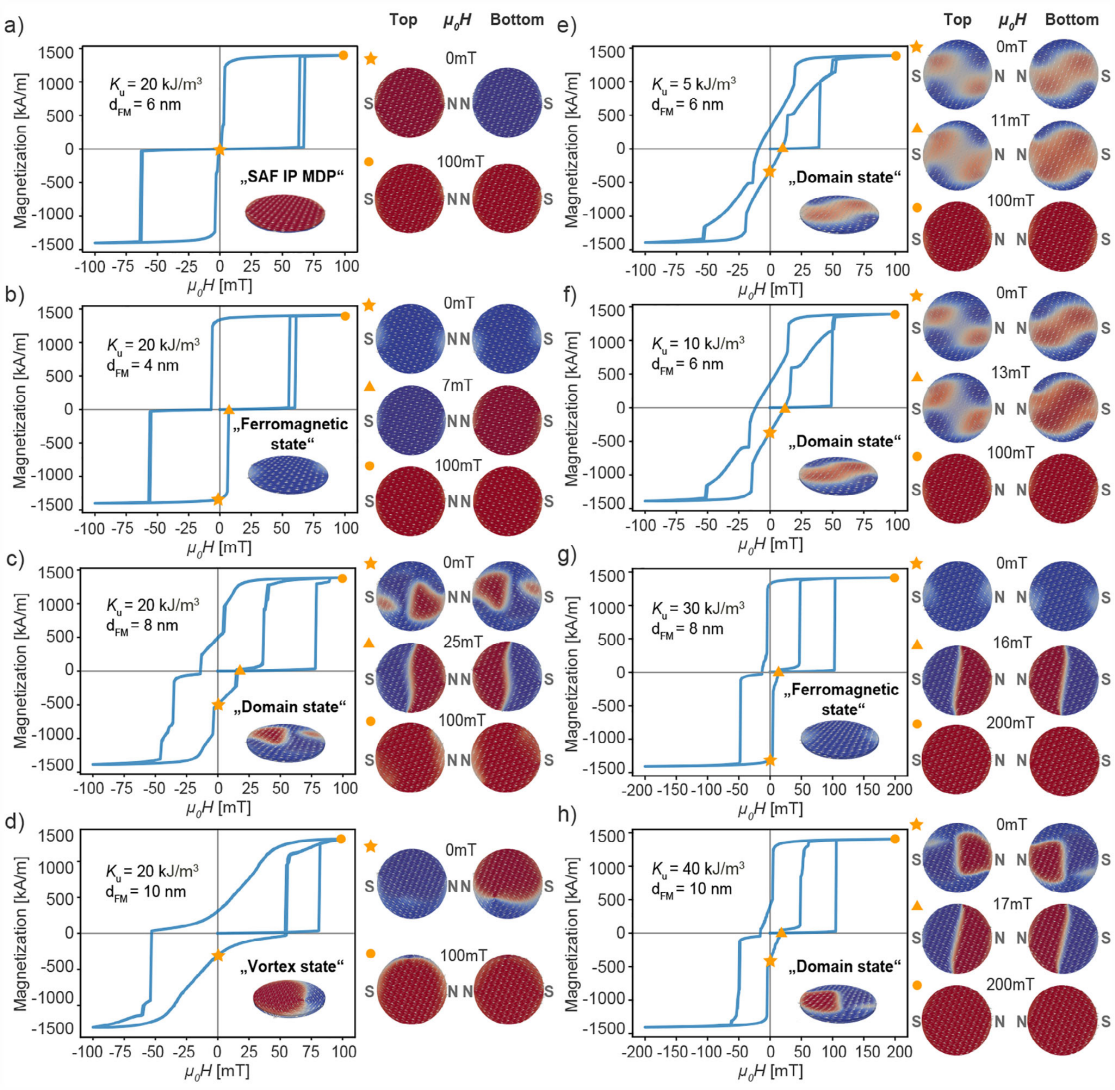
Micromagnetic modeling results for different layer thicknesses and anisotropies illustrating easy‐axis M(H)‐loops together with a 3D image of the magnetic moment distribution of the SAF MDP at zero field shown as an inset and 2D images of top and bottom ferromagnetic layer for different magnetic fields. a–d) micromagnetic modeling results for ferromagnetic layer thicknesses of 4, 6, 8, and 10 nm and an anisotropy kept constant at 20 $\text{kJ}/m^{3}$. e, f) micromagnetic modeling results for anisotropies of 5 and 10 $\text{kJ}/m^{3}$, respectively, and a layer thickness of 6 nm, g,h) results for $K_{u}=30$ $\text{kJ}/m^{3}$, $t_{F}=8$ nm, and $K_{u}=40$ $\text{kJ}/m^{3}$, $t_{F}=10$ nm, respectively.

To reduce the parameter space and find an optimized parameter set to obtain robust SAF MDP properties, micromagnetic modeling was performed. The goal was to find a parameter set leading to desired SAF MDP magnetic properties, such as a sharp and robust switching from the antiferromagnetically aligned ground (AF state) into the ferromagnetic state (F state) in a reasonably small field (<50 mT), and a robust and sharp return to the antiferromagnetic ground state after removal of the field. **Figure** **1a–d** show the obtained easy‐axis M(H) loops for a uniaxial in‐plane magnetic anisotropy $K_{u}$ = 20 ${\mathrm{kJ}/m}^{3}$ and thicknesses of the ferromagnetic layers of 4, 6, 8, and 10 nm, respectively. A magnetization of 1420 kAm^−1^ and an interlayer thickness of 2 nm was used for all calculations presented here. The easy‐axis M(H) loop of the 6 nm thick layers and $K_{u}$ = 20 $\text{kJ}/m^{3}$ (Figure 1a) shows the expected SAF MDP switching behavior. The switching from the AF ground to the F state occurs at a field of 63 mT and is abrupt. Conversely, when ramping down the field the SAF MDP switches back into the AF ground state close to zero field also within a very narrow field range between about 4 and 1 mT. The inset shows a 3D view of the magnetic moments of the SAF MDP. The micromagnetic simulations hence confirm that such an SAF MDP shows the desired turn‐on/turn‐off properties. This also becomes apparent from the magnetic moment distributions shown on the right side of the M(H)‐loop. These show the AF magnetic moment alignment in the top and bottom ferromagnetic layers at zero field and the saturated state at 100 mT. It is noteworthy that for both the AF and F state the magnetic moments deviate slightly from the easy anisotropy axis, with the deviation being largest near the magnetic N and S poles (for the top ferromagnet) on the right and left side, respectively (see arrows). However, note that these micromagnetic simulations did not include effects of temperature.

For a thickness of 4 nm the magnetostatic interaction between the two F layers is reduced. Consequently, the minor‐hysteresis loops are less shifted away from the center (Figure 1b). For a thickness of 8 nm, Figure 1c, the M(H)‐loop no longer shows a sharp switching behavior. The magnetic moment distributions inside the top and bottom ferromagnetic layers depicted on the right side reveal that domains are formed inside the ferromagnetic layer.

For a ferromagnetic layer thickness of 10 nm (Figure 1d), the M(H)‐loop differs considerably from an ideal SAF MDP loop such as the one depicted in a. As expected for a sufficiently thick magnetic layer, a vortex state develops at zero field (see top row of the magnetic moment distribution in the disks), which can be annihilated at a sufficiently high applied field. Figure 1e,f then show the M(H)‐loops and magnetic moment distributions for SAF MDPs with a ferromagnetic layer thickness kept constant at 6 nm, but anisotropy values for 5 and 10 $\text{kJ}/m^{3}$, respectively, and show M(H)‐loops comparable to an anisotropy of 5 $\text{kJ}/m^{3}$ obtained for a ferromagnetic layer thickness of 8 nm and an anisotropy of 20 $\text{kJ}/m^{3}$. Again domains are formed, however, for these lower anisotropies with wider walls and consequently with a local magnetic moment alignment further away from the easy‐magnetization axis. Domain formation becomes more favorable because of the lower domain wall energy given by $\sigma_{w}$ = 4$\sqrt{K_{u}A}$,^[^ ^15^ ^]^ where A is the exchange stiffness. Figure 1g then reveals that the switching of the loop again becomes sharper as the anisotropy of 8 nm‐thick ferromagnetic layers is increased to 40 $\text{kJ}/m^{3}$ and for most fields domains are suppressed by the increased domain wall energy. Figure 1h finally shows the M(H)‐loop and micromagnetic states obtained for 10 nm‐thick ferromagnetic layers and an anisotropy $K_{u}=40$ $\text{kJ}/m^{3}$. The increased anisotropy now suppressed the vortex but a domain state is formed, very much alike the micromagnetic states which develop in the 8 nm, $K_{u}=20$ $\mathrm{kJ}/m^{3}$ SAF MDP system. Note that, as revealed by our micromagnetic modeling, SAF MDPs can be designed to be in a demagnetized state initially to form a stable suspension, but then can be turned‐on by application of a relatively small (30 mT) field, which breaks the antiferromagnetic arrangement of the two ferromagnetic layers. If the coupling field of the two ferromagnetic layers is designed to be weaker than that needed to overcome the anisotropy barrier, the SAF MDP remain in a switched‐on state even after field removal. Such an “on‐state” leads to a rapid coagulation of the SAF MDPs into larger agglomerates, which destabilize the particle suspension and thus facilitate a successive removal of the particles from the liquid.

Taken together, the micromagnetic simulations reveal that the interplay of the different relevant magnetic energy terms can lead to vortex or domain states such that the M(H)‐loop deviates from an ideal SAF MDP behavior. Generally, an M(H)‐loop that maximizes the hysteretic loss can only be obtained by a careful selection of the geometrical and magnetic parameters of the SAF system with the unaixial anisotropy being a particularly critical parameter documenting the importance of micromagnetic modeling.

### 2.2 Achieving a Suitable Uniaxial in‐Plane Anisotropy

**Figure 2:**
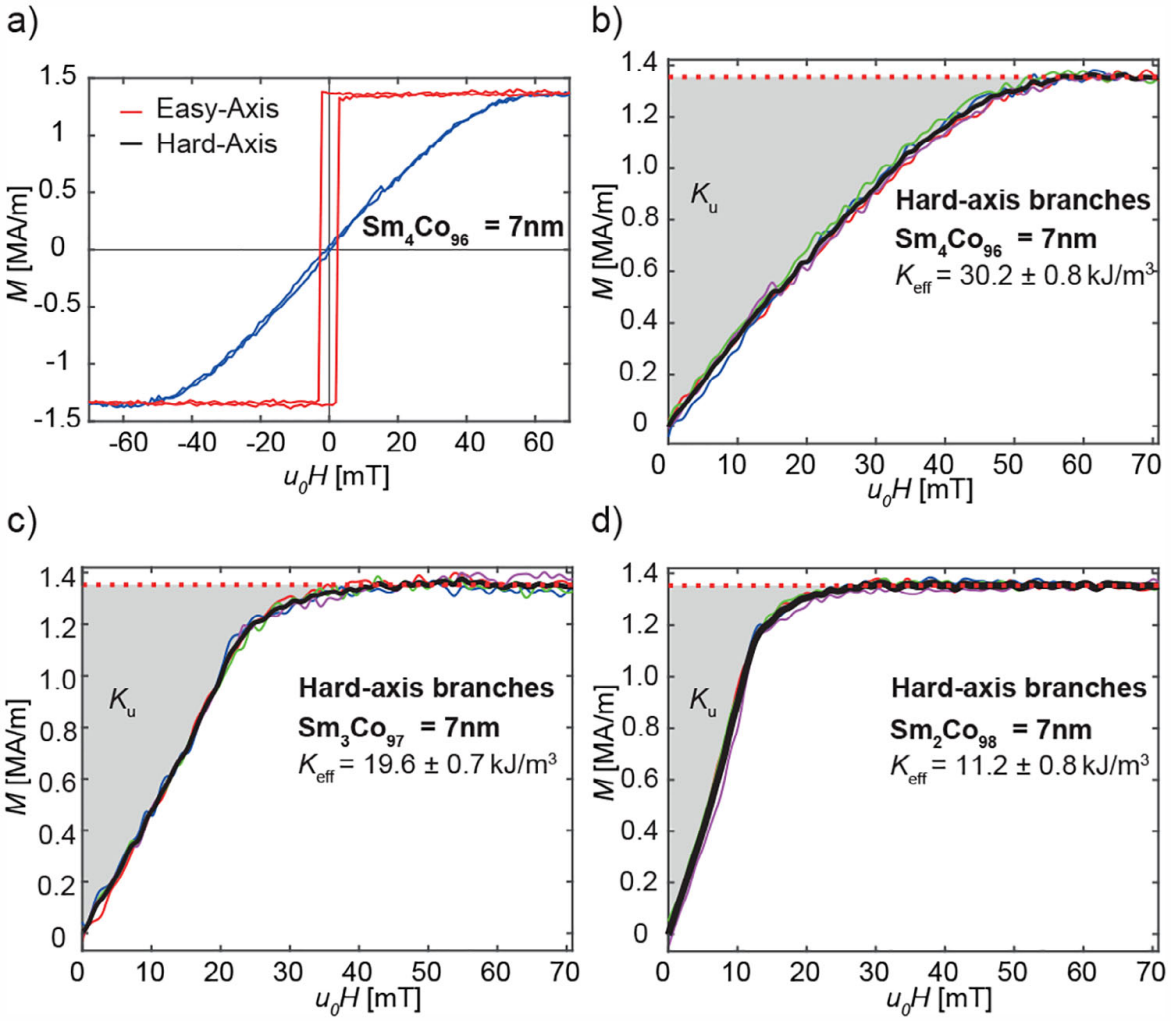
a) Easy‐ and hard‐axis M(H)‐loop obtained by Kerr microscopy for a ${\mathrm{Sm}}_{x}$ ${\mathrm{Co}}_{100-x}$ layer with 4 % Sm content and 7 nm thickness sputter‐deposited in a 40 mT magnetic field. b,c) hard‐axis M(H)‐loops for films with 4 %, 3 %, and 2 % Sm content, respectively, along with the average of the four curves represented by the black line.

As our micromagnetic simulations have revealed, a suitable uniaxial in‐plane magnetic anisotropy is prerequisite for the desired SAF MDP switching properties. Uniaxial in‐plane magnetic anisotropy can be achieved by various methods, among them sputtering onto obliquely deposited seed layers,^[^ ^37^, ^38^, ^39^, ^40^, ^41^ ^]^ oblique deposition of the magnetic layer^[^ ^42^, ^43^, ^44^ ^]^ or deposition in an applied field.^[^ ^45^, ^46^ ^]^ Large in‐plane anisotropies up to 100 ${\mathrm{kJ}/m}^{3}$ were reported by Magnus et al.^[^ ^47^ ^]^ for an in‐field‐deposition of amorphous ${\mathrm{Sm}}_{x}$ ${\mathrm{Co}}_{100-x}$ alloys with x=2 to 35 at. %. In the SAF MDP, we aimed for an anisotropy of only 20 $\text{kJ}/m^{3}$, which according to the work of Magnus et al.^[^ ^47^ ^]^ could be presumably obtained at Sm contents below 4 at. %. A 5 nm‐thick amorphous ${\mathrm{Al}}_{70}{\mathrm{Zr}}_{30}$ seed layer was used for oxidation protection of the bottom surface of the ferromagnetic ${\mathrm{Sm}}_{x}$ ${\mathrm{Co}}_{100-x}$ layer and also to promote an amorphous growth for low Sm contents. To obtain an anisotropy of 20 $\text{kJ}/m^{3}$, as used in our micromagnetic models, a 7 nm thick ${\mathrm{Sm}}_{x}$ ${\mathrm{Co}}_{100-x}$ films with x = 2 to 4 at. % were deposited onto 5 nm‐thick ${\mathrm{Al}}_{70}$ ${\mathrm{Zr}}_{30}$ seed layers and covered with an identical oxidation protection layer, in an applied field of 40 mT. **Figure** **2a** shows a hard‐axis (blue line) and easy‐axis (red line) M(H)‐loops measured by Kerr microscopy on a ${\mathrm{Sm}}_{x}$ ${\mathrm{Co}}_{100-x}$ layer with 4 % Sm content. Note that the ferromagnetic layer thickness has been increased from the 6 nm used in the micromagnetic modeling to 7 nm to compensate for the observed loss of magnetic moments near the CoSm/AlZr interfaces, i.e., a dead layer formation. The saturation magnetization was determined by Vibrating Sample Magnetometry. The linear and hysteresis‐free hard‐axis loop confirms a Stoner‐Wohlfarth rotatory magnetization process.

The hard‐axis M(H) branches from 0 to 70 mT, 70 to 0 mT, 0 to ‐70 mT, and ‐70 to 0 mT together with the average of the four curves (black line) for films with 4, 3 and 2 % of Sm content, respectively are shown in Figure 2b–d. The anisotropy $K_{u}$ was obtained from the area enclosed between the averaged (black) curve and the y‐axis (grey area). For the films containing 4, 3, and 2 % Sm anisotropies of (30.2±0.8) $\text{kJ}/m^{3}$, (19.6±0.7) $\text{kJ}/m^{3}$, and (11.2±0.8) $\text{kJ}/m^{3}$ were found. ${\text{Co}}_{\text{97}}$ ${\mathrm{Sm}}_{3}$ film has an anisotropy that is very close to the 20 $\text{kJ}/m^{3}$ for which our micromagnetic modeling has revealed an ideal SAF MDP switching behavior (see Section 2.1).

### 2.3 Scalable Microfabrication and SAF MDP Characterization

**Figure 3:**
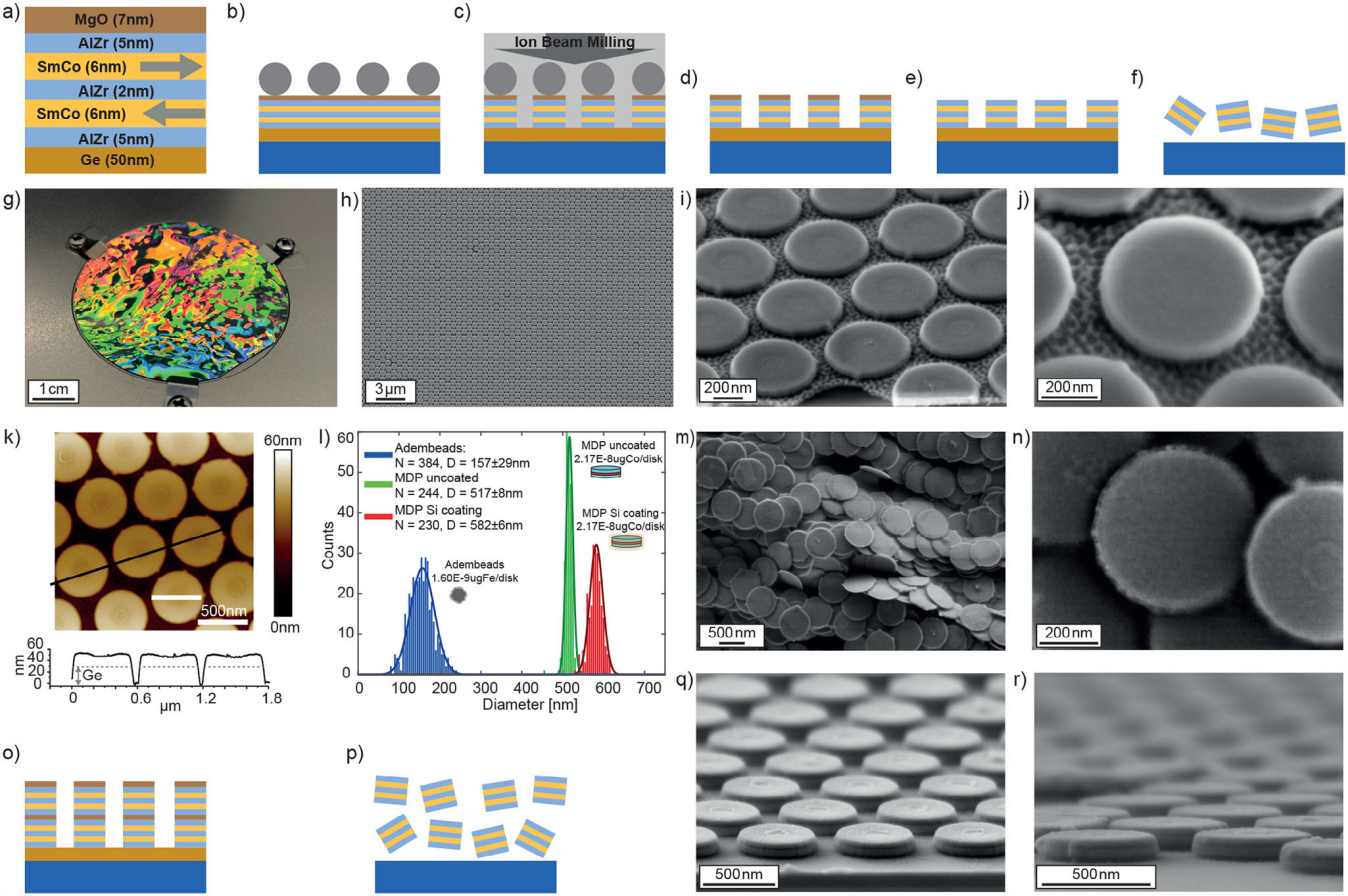
a) A schematic illustration of the in‐plane SAF thin film multilayer stack b) A sacrificial layer of Ge was evaporated on the Si substrate prior to the sputter deposition of the multi‐layer stack. Following PS beads self‐assembly on top of the multi‐layer, the sample was treated with oxygen plasma to separate the PS beads from each other. c) Ion Milling was applied to remove the multilayer stack between the PS beads and define the circular islands. d) PS beads were removed by applying 5min ultrasound in pure water. e) The top sacrificial MgO layer was etched away by a 30 min treatment with citric acid. f) The SAF MDP were released by etching the Ge layer in $H_{2}O_{2}$ for 1.5 h yielding a SAF MDP mass of 130μg per 2‐inch wafer. g) Photograph illustrating that the periodic hexagonal packed ordering of the PS beads on top of the 2‐inch wafer create a diffraction pattern. h–j) SEM images of SAF MDP still attached to the Si wafer. k) AFM height map of SAF MDP attached to the wafer. l) Size distribution of commercially available iron oxide supraparticles (Adembeads), uncoated and Si‐coated SAF MDPs. m) and n) SEM images of released SAF MDP. o–r) Scaling up the fabrication by stacking two SAF multi layers separated by a 7 nm MgO layer. Etching the Ge bottom sacrificial layer as well as the MgO intermediate sacrificial layer releases the particles yielding an estimated total particle mass of 260μg per wafer. SEM image using a tilt stage showing the lateral view on a double stack of SAF MDP.

For the microfabrication of SAF MDPs from the sputter‐deposited CoSm/AlZr multilayers (see **Figure** **3a**), a polystyrene‐based lithography approach adapted from Armstrong and O'Dwyer^[^ ^48^ ^]^ was employed. The CoSm/AlZr multilayers were sputter‐deposited on 2‐inch Si wafers with a 50 nm‐thick Ge sacrificial layer. The latter enabled the detachment of the patterned disks from the wafer after completion of the fabrication process. To induce a well defined uniaxial in‐plane magnetic anisotropy, the sputtering was carried out in field (see Section 2.2). The multilayers were capped with a top sacrificial layer of 7 nm MgO. On top of the MgO layer, polystyrene (PS) beads were self‐assembled on a liquid‐air interface and used as a hard mask for a successive ion etching process. For the hard mask, carboxylate‐modified PS beads with a diameter of 660 nm were self‐assembled at the water‐air interface,^[^ ^32^, ^49^ ^]^ creating a highly ordered hexagonal packed monolayer (Figure 3g)). The obtained long‐range order of the PS beads over a full 2‐inch Si wafer becomes apparent from the diffraction pattern easily visible to the nacked eye, whereas the excellent local hexagonal ordering was confirmed using electron microscopy (see Figure S1, Supporting Information). Oxygen plasma etching was then employed to reduce the PS bead diameter to 500 nm and obtain a sufficiently large distance between the beads for the successive Ar milling to remove the multilayer coating between the beads. Afterwards the PS beads were removed by sonication in water (Figure 3d), yielding circular disk‐shaped pillars still attached to the wafer (Figure 3h‐j). Atomic force microscopy (AFM) of the MDPs on the wafer revealed a smooth top layer with less than 5 nm roughness. The height of the MDPs on the wafer (Figure 3k) was determined to be approximately 50 nm, which is higher than the overall thickness of the magnetic multilayer (33 nm). This is because the ion‐milling process was continued into the Ge sacrificial layer to ensure complete milling of the bottom AlZr layer. Finally, the MgO top sacrificial layer was removed using citric acid and the Ge bottom sacrificial layer was dissolved in a final step to release the SAF MDPs from the wafer into suspension. SEM of the released MDPs indicated high uniformity of the MDPs (Figure 3m,n). Minor residuals of the polystyrene mask were visible as a ring on top of the particles (see SEM image in Figure 3i)), which however, may be eliminated through an extended etching duration of a top sacrificial layer. The yield per wafer was a MDP mass of 130μg per 2‐inch wafer, equivalent to 66 % of the theoretically possible maximum, assuming a perfect mask. The released MDPs show uniform size and shape (Figure 3m,n), and a narrow size distribution (Figure 3l) compared to commercially available Adembeads (Ademtech, 33600 Pessac, France) supraparticles which serve as the gold standard in magnetic separation. Adembeads (Figure S1, Supporting Information Magnetization at saturation: ≈40 emu/g, Iron oxide content: ≈70 %, surface area 10I $m^{2}/g$) have successfully been employed by Kang et al.^[^ ^50^ ^]^ in their seminal work on in vivo blood purification.

Note that the fabrication method presented here can be adapted (see Figure 3o–r), enabling the stacking of the multilayers with intermediate sacrificial MgO layer. Lateral inspection of the stacked MDPs in SEM using a tilt stage shows a demarcation line between the two particles (Figure 3q,r) with otherwise almost surface‐normal side walls of the particles. In this way, a doubling of the number of MDPs to 260μg per 2‐inch wafer can be achieved without detectable compromises in MDP quality.

### 2.4 SAF MDP Composition, Stability, and Magnetic Properties

**Figure 4:**
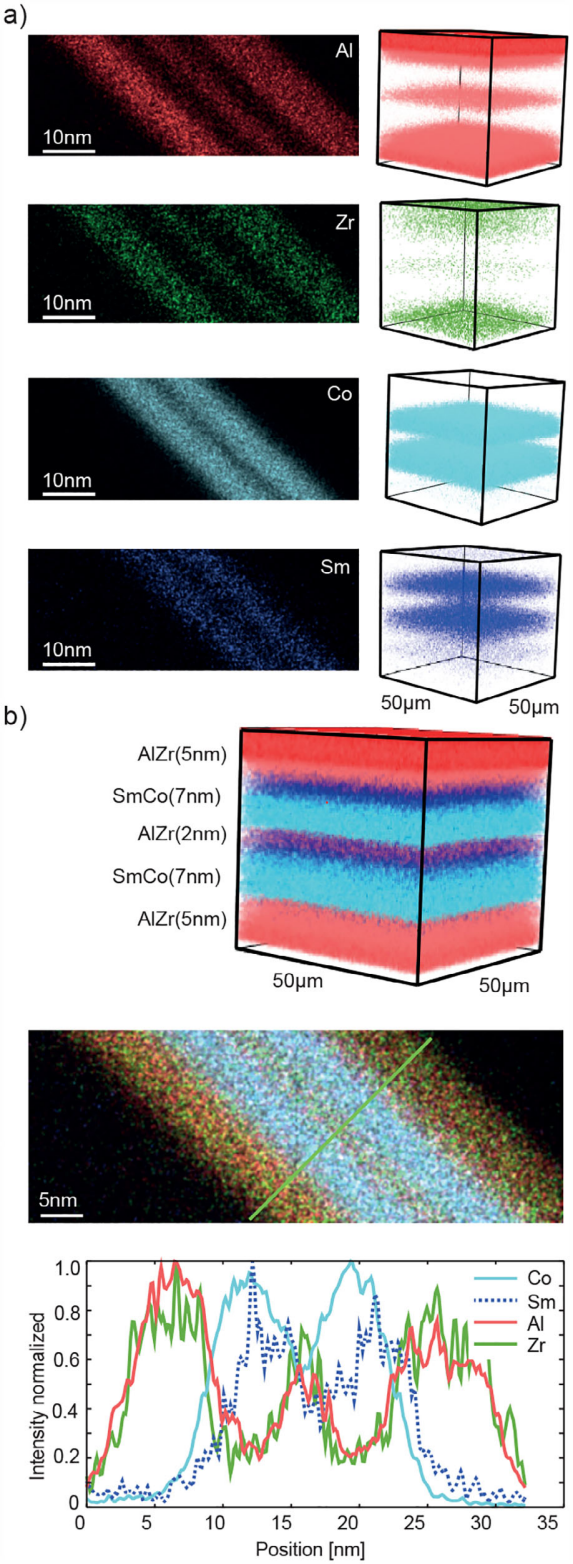
a) SAF MDP elemental composition displayed based on an EDXS elemental map of a FIB cut TEM lamella (left) and TOF‐SIMS (right). b) a 3D compositional analysis based on ToF‐SIMS data (top) and EDXS map with line profiles (bottom).

Following microfabrication, the layered architecture of the magnetic and non‐magnetic layers of the SAF MDPs was confirmed using a Focused‐Ion Beam (FIB) cut lamella imaged by Scanning Transmission Electron Microscopy with Energy‐dispersive X‐ray Spectroscopy (STEM‐EDXS) elemental mapping, and a Time of flight ‐ Secondary Ion Mass Spectrometry (TOF‐SIMS) depth profile of the multilayer structure on the substrate (see **Figure** **4**). In the EDXS maps of the TEM lamella, the two magnetic layers containing the Co and Sm separated by a thin non‐magnetic layer and sandwiched between two non‐magnetic Al/Zr capping layers are clearly visible. Segregation in the z‐direction, i.e., a non‐homogeneous mixing, of the Co and Sm in the magnetic layers is observed. This segregation has been further confirmed by TOF‐SIMS of the multilayer on the wafer. Such segregation is well in line with previous observations for rare‐earth elements in iron.^[^ ^51^ ^]^

The elemental composition of the as‐synthesised SAF MDPs was quantitatively analysed by inductively coupled plasma mass spectrometry (ICP‐MS) as well as semi‐quantitative EDXS measurements and was in good agreement with the theoretical composition (less than 3 % deviation, see Figure 6a) for theoretical and measured values). Based on the ICP‐MS measurement, the Sm content was determined as 2 % and the Co content as 64 %, which are in reasonable agreement with the theoretical value of 3 % and 61 %, respectively, and an overall non‐magnetic fraction of 33 %. Taken together, these results attest to the high compositional (quantified based on ICP‐MS) and architectural (assessed by EDXS and TOF/SIMS) control of the deposited layers during the sputtering process, allowing the precise control needed for the fabrication of magnetic designer nanoparticles with performance‐optimized properties, e.g., according to micromagnetic simulations.

**Figure 5:**
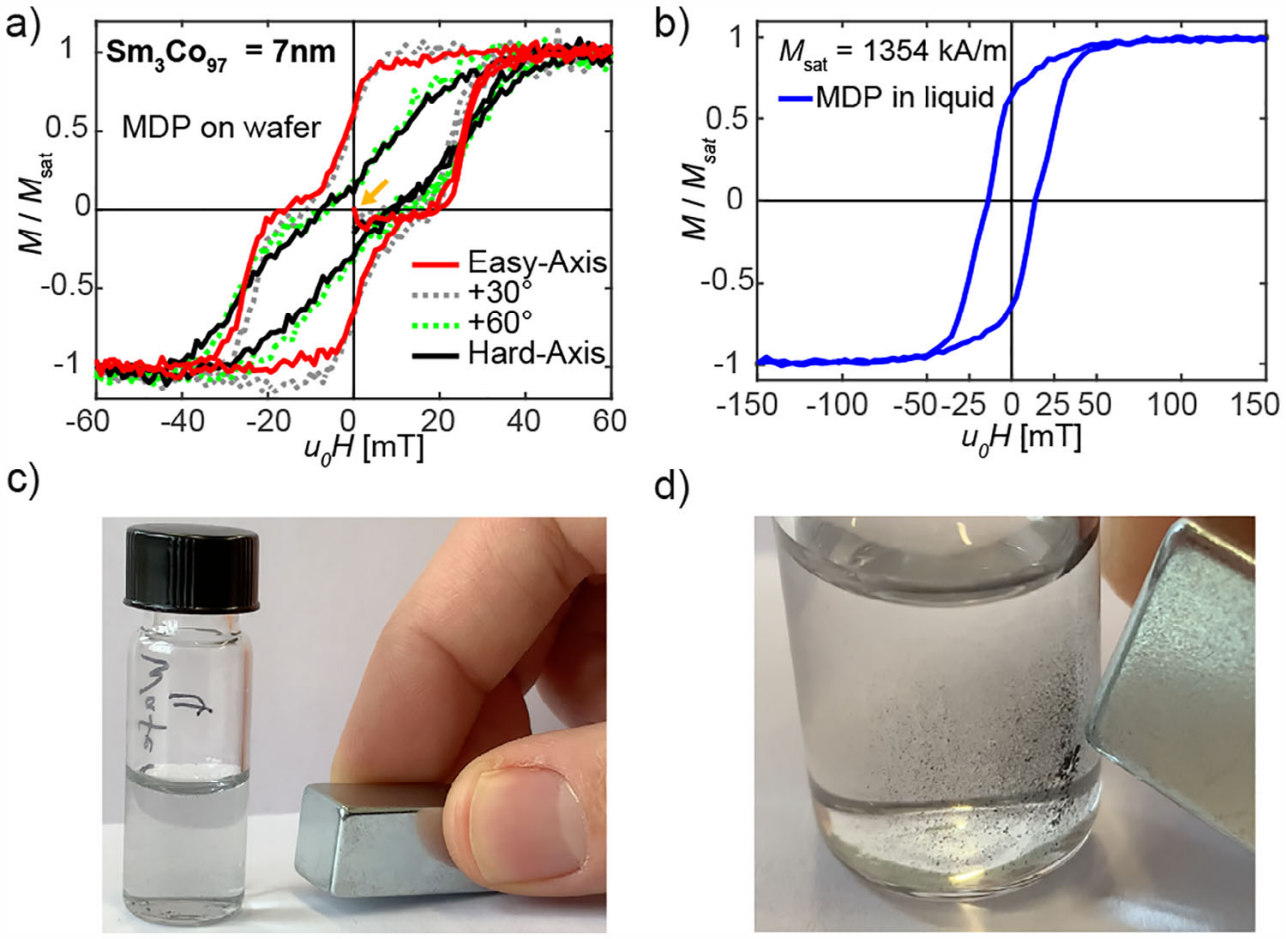
a) Kerr M(H)‐loop of MDP while still attached to the wafer, including the virgin curve. Prior to the measurement, the particles were demagnetized. b) VSM M(H)‐loop of suspension of SAF MDP in water. A saturation magnetization of 1354 $\text{kA}/m^{1}$ was measured for a thin film multilayer sample prior to the fabrication of the SAF MDPs. c) Stable suspension of SAF MDPS in water, maintaining a non‐agglomerated state when not exposed to an external magnetic field, and in d) exhibiting a ferromagnetic state when subjected to an external magnetic field. Magnet has a square crossectional area of $1\;{\mathrm{cm}}^{2}$.

**Figure 6:**
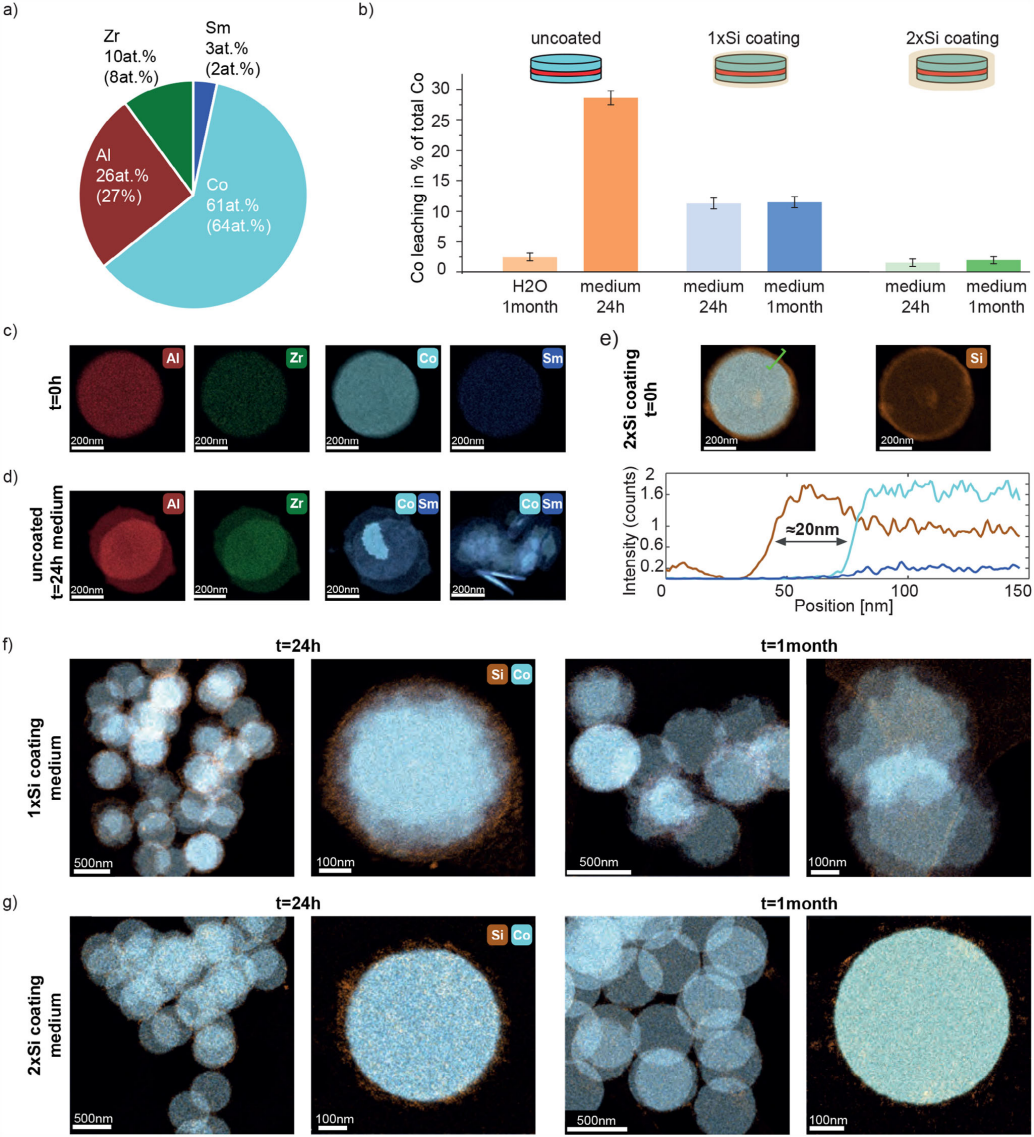
a) Elemental composition of SAF MDPs determined using ICP‐MS (theoretical values in brackets). b) Co leaching expressed as a percentage of total Co. c,d) EDXS elemental maps of Al, Zr, Co and Sm for uncoated SAF MDPs before (t=0) and after incubation in cell medium for 24h. e) EDXS elemental maps of native Si‐coated SAF MDP along with a line profile indicating a silica shell thickness of around 20 nm. f,g) EDXS elemental maps shown for Si‐coated SAF MDPs with single (1x) and double (2x) Si‐layers after 24h and 1 month in cell medium, respectively. Two different magnifications are shown.

After confirming the correct structure and composition of the SAF MDP, their magnetic properties were analyzed for the MDPs still attached to the wafer as well as in suspension after their detachment from the substrate by dissolution of the sacrificial Ge layer. The results are shown in **Figure** **5**a,b. The easy and hard axis M(H)‐loops acquired by Kerr microscopy on an SAF MDP with 7 nm‐thick ${\mathrm{Sm}}_{3}$ ${\mathrm{Co}}_{97}$ ferromagnetic layers having an anisotropy of 19.5 $\text{kJ}/m^{3}$ (see Section 2.2) are displayed as the solid red and black lines in Figure 5a, respectively, for the MDPs still attached to the wafer substrate. Following observations need to be discussed:

The easy axis loop shows a switching from the AF ground to a F state for an applied field of about 30 mT, that is slightly smaller than that expected from the micromagnetic modeling work (see Figure 1a for comparison). This is a quite common observation when comparing modelled to measured loops, as the modelling does not include effect of temperature, or defects in the magnetic layer, which both facilitate magnetization reversal. Further, the SAF MDP shows a remanence at zero field, i.e., does not switch back completely into the AF ground state but requires the application of a negative field when returning from positive saturation. This can arise from the formation of domains and from a pinning of their walls, or alternatively from an AF coupling, which may be reduced by either a slightly smaller ferromagnetic layer thickness or a reduced magnetic moment, a thicker intermediate AlZr layer, or simply from the variations in diameters for different particles (see Figure 3l). All aforementioned cases and combinations thereof would widen the switching field distribution and may explain the remanence. The hard‐axis loop is reminiscent of a Stoner‐Wohlfarth hard axis loop, but shows a small hysteresis. We attribute this to variations of the average anisotropy for different particles, or again, to pinning occurring from spatial variations of the magnetic properties inside the ferromagnetic layers.

The M(H)‐loops of the fabricated SAF MDP deviate from those of the modelled loops. The observed remanence of the SAF MDP after removing a previously applied field may lead to a destabilization of a suspension made from such particles. We hence investigated whether the SAF MDP (still attached to the wafer) could be demagnetized by application of an oscillatory field with a decaying amplitude. Because for particles in suspension, it would not be possible to apply the field exactly along the easy axes of all particles, the demagnetization field for the particles on the wafer was applied along the easy axis, 30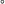 and 60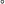 away from it and along the hard‐axis. All juvenile M(H)‐curves started from zero magnetic moment (see orange arrow in Figure 5a) confirming that the demagnetization process works irrespective of the alignment of the demagnetization field with the anisotropy axes of the particles. We can thus conclude that the SAF MDP can be brought into a zero magnetic moment state by a suitable demagnetization procedure such that attractive inter‐particle forces can be suppressed and a stable suspension can be obtained. Moreover, having a remaining magnetic moment after field removal (as for example observed by the micromagnetic modelling work performed on samples with 4 nm thickness of the ferromagnetic layer) may prove to be advantageous for particle‐based magnetic separation applications, because once the SAF MDP in a suspension become magnetized, the particles would remain in an F state even after removal of the field, which would then lead to particle coagulation facilitating their effective removal from the liquid.

The M(H)‐loop obtained for the SAF MDPs in liquid is depicted in Figure 5b. It is noteworthy that the typical shape of an SAF MDP easy‐axis loop, as observed for the field aligned with the easy axis for particles attached to the wafer, is no longer apparent. However, the remanence is about of the same size. The loop for the particles in suspension can be understood as an average of easy‐ and hard‐axis loops (and loops acquired for field direction angles between 0 and 90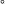 away from the easy‐axis), because in suspension the SAF MDP do not necessarily align their easy‐axes with the field. Note that the SAF MDPs in suspension reach a very high magnetic moment, i.e., are driven to their F state, by application of a very moderate field and exhibit a saturation magnetization of 1354 kA/m.

Figure 5c,d then show suspension of the particles in zero field and after putting the edge of a permanent magnet close the glass tube, respectively. After applying the field (and field gradient), the SAF MDP coagulate into visible more macroscopic particle assemblies that become visible for the naked eye and move towards the edge of the magnet confirming that the separation process with our SAF MDP is successful.

Following assessment of the magnetic properties, the SAF MDPs were characterized in terms of chemical and colloidal stability, as both of the aforementioned properties are imperative for successful biomedical application. To assess the colloidal properties of the as‐synthesized SAF MDPs in physiologically relevant dispersants, the particles were re‐suspended in Milli‐Q water or cell culture media, respectively. Colloidal stability was sufficient for biomedical use with suspensions remaining stable for several days (see Figure 5c,d). Particles could be readily separated and resuspended using low‐power sonication. The MDPs showed high colloidal and chemical stability in water without significant ion leaching over time (<2 % over 1 month). Interestingly, however, we noticed significant leaching (<30 % within 24 h) of cobalt ions from the particles in cell culture media (see **Figure** **6**b). Even more interestingly, based on ICP‐MS and EDXS (see Figure 6b–d), it becomes evident that the samarium (as well as the Al and Zr) remained in the solid phase, indicating that cobalt selectively leached out (comparing Figure 6c,d). This finding has two important consequences; i) the leached cobalt significantly diminishes the magnetic properties of the SAF MDPs in cell culture media, and ii) the leached cobalt ions may cause cytotoxicity. Thus, in order to prevent cobalt leaching, MDPs were encapsulated in silica shells using tetraethyl orthosilicate (TEOS) (see Figure 6e,g). Silica was selected due to its superior hermetic properties when compared to PEG^[^ ^52^, ^53^ ^]^ and scalable application, as opposed to gold.^[^ ^54^ ^]^ Thin silica coatings of <10 nm coating thickness were only partially effective (Figure 6f). However, encapsulation with 20 nm silica of SAF MDPs largely prevented cobalt leaching (< 1.5 % over 24 h) and the SAF MDPs retained their excellent magnetic properties even in cell culture media for at least one month (<2 % Co leaching after 1 month in cell culture media as shown in Figure 6g). These data highlight the importance of a protective shell fully encapsulating the SAF MDPs. Both the Al/Zr oxide as well as the silica capping offer versatile platforms for surface modification using, e.g., silanes. This is shown exemplary using TEOS/(3‐Aminopropyl‐triethoxysilane (APTES) on Al/Zr oxide capped SAF MDPs.

### 2.5 Silica‐Encapsulation and Surface Functionalization

**Figure 7:**
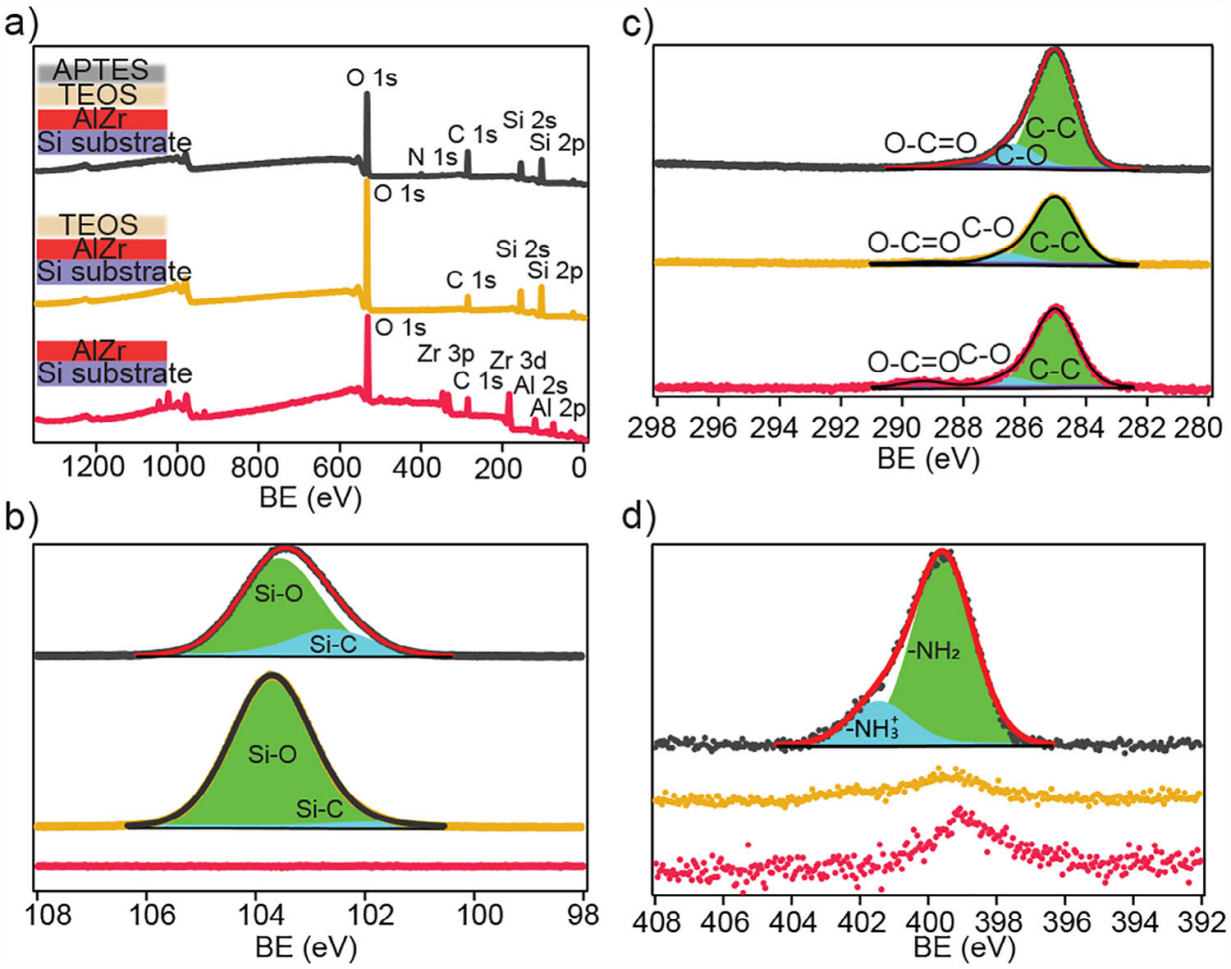
a–d) XPS spectra of uncoated (red lines), silica‐encapsulated (yellow lines) and APTES‐functionalized samples (black lines).

To demonstrate the straightforward surface functionalization of the SAF MDPs, the silica‐encapsulated particles were surface functionalized with APTES, a versatile surface anchoring molecule with a primary amine, offering a versatile precursor for further functionalizations, e.g., using EDC/NHS coupling. X‐ray photoelectron spectroscopy (XPS) analysis of uncoated, silica‐encapsulated, and silica‐APTES‐functionalized SAF MDPs was employed to monitor the surface functionalization (**Figure** **7**).

According to the survey spectra shown in Figure 7a, the surface of uncoated particles contains Al, Zr, and C. In addition to the Al and Zr expected as a result of the metal (oxide) capping, the C 1s spectrum of uncoated MDP Figure 7b, indicates three components peaks of C‐C/C‐H, C‐O, and C = O positioned at 285.0, 286.5, and 289.2 eV, respectively.^[^ ^55^, ^56^ ^]^ Neither Si nor N are detectable in the uncoated samples. Silica‐encapsulation of the SAF MDPs using TEOS expectedly alters the XPS signal. As seen in Si 2p spectra in Figure 7, the silanization process results in the formation of siloxane groups on the surface, leading to appearance of Si‐O and Si‐C peaks at 103.7 and 102.9 eV, respectively.^[^ ^55^, ^57^ ^]^ The addition of APTES further increases the intensity of the aforementioned components. The intensity increase of C 1s components, when comparing uncoated, silica‐encapsulated, and APTES‐functionalized SAF MDPs, results from the presence of more organic groups on the surface. With APTES treatment of the surface, an additional N 1s peak appears (Figure 7b,d). The strong N peak in the N1s high resolution spectrum is related to C‐amine/protonated amine groups of APTES and fitted with NH_2_ and NH_3_^+^ at 399.5 and 401.4 eV, respectively^[^ ^55^, ^56^, ^58^ ^]^

Taken together, these XPS data show the successful silica‐encapulation and subsequent surface functionalization of the SAF MDPs with primary amines. The primary amine of APTES enables further functionalization using e.g., amine reactive crosslinkers, following strategies similar to the work by Asad et al.^[^ ^59^ ^]l^

### 2.6 SAF MDP Cytocompatibility

**Figure 8:**
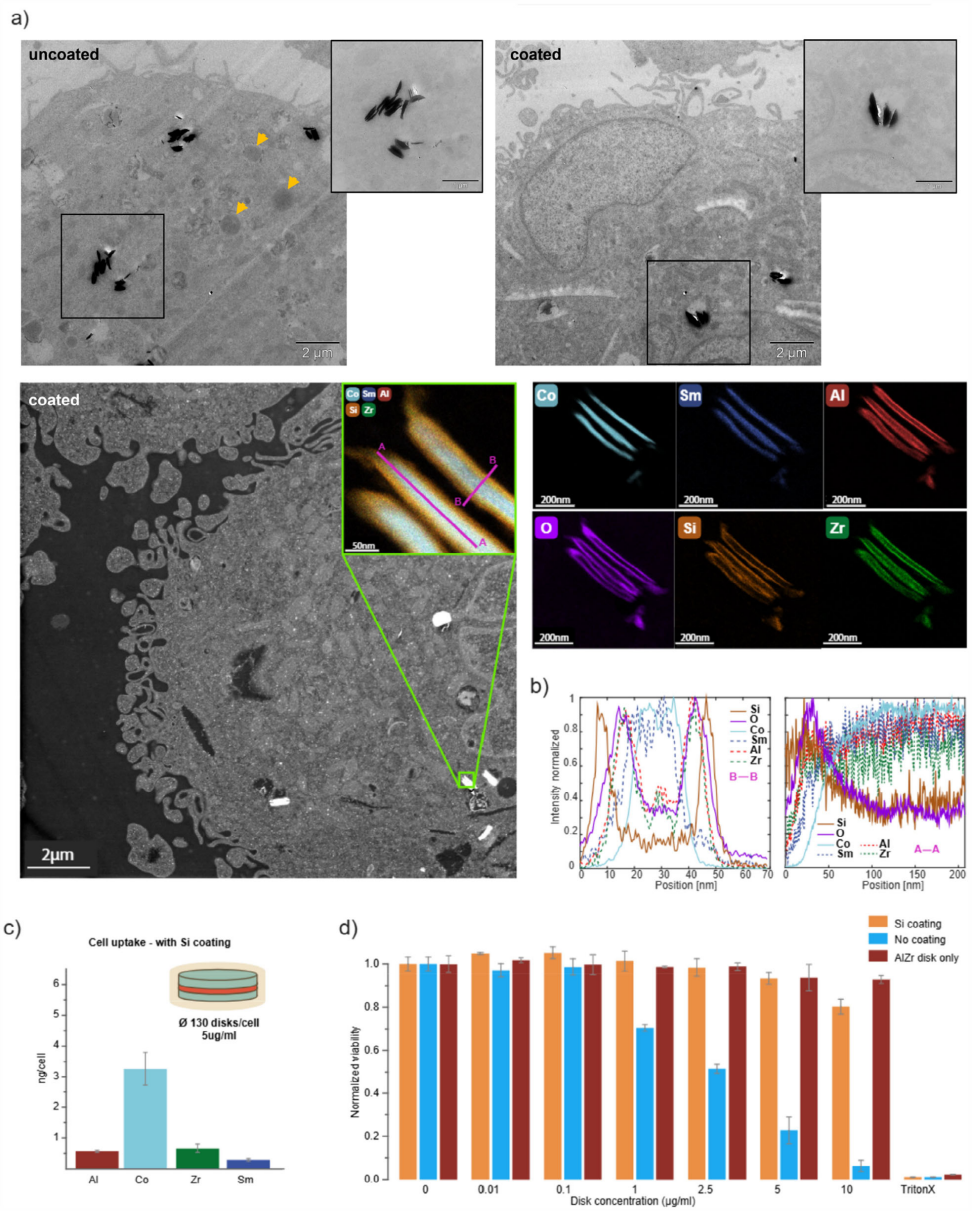
Cytocompatibility of SAF‐MDPs. a) Transmission electron micrographs of uncoated and Si‐coated SAF‐MDPs. Orange arrows indicate autophagy. High‐angle annular dark field (HAADF)‐STEM micrographs of intracellular Si‐coated SAF‐MDPs and elemental maps aquired using EDXS. b) Line profiles of elemental content assessed using EDXS. c) Cellular uptake of SAF MDP measured by ICP‐MS. d) Human macrophage cell viability as a function of uncoated, Si‐coated SAF MDPs as well as AlZr disks concentration and exposure for 24 h.

To assess the compatibility of the SAF MDPs, cell culture studies were performed using human macrophages, representing most relevant target cells for such nanoparticles.^[^ ^60^ ^]^ Silica‐encapsulated and non‐encapsulated MDPs were administered to human macrophages at doses up to 10 µg/mL and incubated for 24 h. TEM images of epoxy embedded, thinly sectioned macrophages revealed that the MDPs were readily internalized by the cells and contained in membrane‐bound sub‐cellular compartments with the characteristic size of endo/lysosomes (see **Figure** **8**). Within the individual compartments, a preferred alignment and stacking of the MDPs could be observed. Note that MDPs can be found exclusively in the cytoplasm and did not enter in the nucleus. Macrophages exposed to uncoated SAF MDPs show signs of autophagy and cytotoxicity, while cells exposed to Si‐coated SAF MDPs show intact ultrastructure. Based on EDXS elemental maps of intracellular SAF MDPs, it can be observed that the non‐silica‐encapsulated SAF MPDs undergo peripheral oxidation as seen in Figure 8a, and regions devoid of cobalt can be detected even towards the central regions of the MDPs, in line with the data on MDPs suspended in cell culture media. EDXS line scans indicate that the cobalt devoid regions are richer in oxygen compared to the cobalt‐containing regions, indicating oxidation of the samarium. These data on non‐encapsulated MDPs suggest oxidation and Co leaching also in cell cultures, analogous to experiments where MPDs were exposed to cell‐free culture media for 24 h or longer.[Fig adhm202500616-fig-0009]

**Figure 9 adhm202500616-fig-0009:**
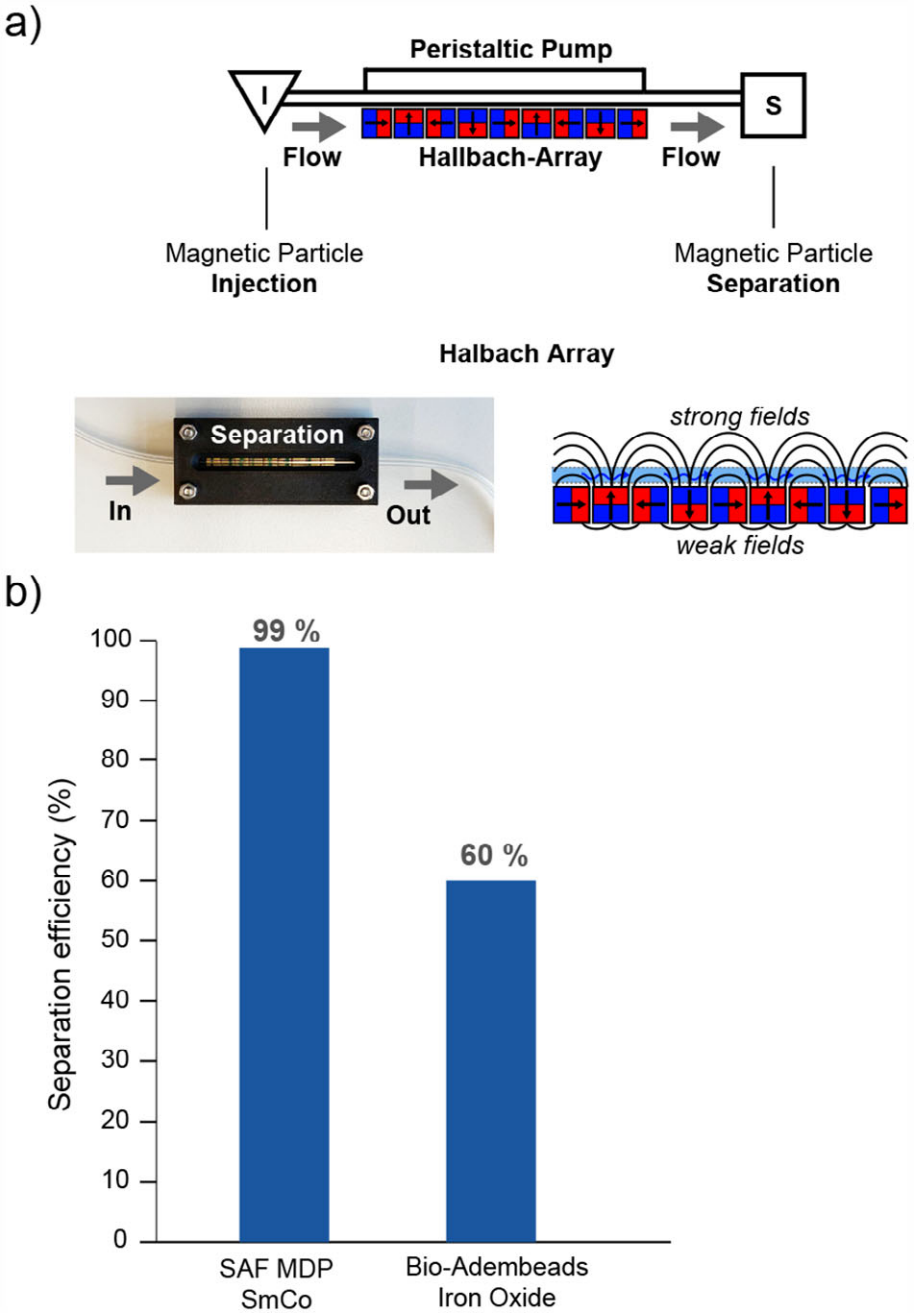
Study of the magnetic particle separation. a) Experimental setup consisting of injection point and separation point connected via silicon tubing and two Halbach array magnets. b) SAF MDPs exhibit superior separation efficiency (99 % removed) compared to commercial Adembeads (60 % removed) under the same high flow rate of 1 mL/min.

The silica‐encapsulation effectively prevents oxidation and loss of Co, as evident from the EDXS mappings and the elemental distributions (see Figure 8b). Oxygen is constrained to the outermost Si layer and the AlZr capping layers, and the MDPs are largely devoid of oxygen in the CoSm‐containing regions (as seen based on the line‐scan A‐A, in Figure 8b). In the EDXS line scan normal to the disk (B‐B), the spatial separation of the ${\text{SiO}}_{2}$, the ${\text{AlZrO}}_{x}$, and the CoSm can be seen. Well in line with the TOF/SIMS data, segregation of the Co and Sm can again be observed. These distinct differences in behavior for the non‐silica‐encapsulated and the silica‐encapsulated MDPs are further supported by viability data.

While for the non‐silica‐coated MDPs, surprisingly high cytotoxicity (≥30 % decrease in metabolic activity for administered doses of 1μg/mL, corresponding to 260 MDPs per cell) values were found in macrophages, cell compatibility was significantly improved for the silica‐encapsulated MDPs (100 % viability as shown in Figure 8d). Interestingly, our viability data for the non‐encapsulated MDPs were similar to data from previous work by Yu et al.^[^ ^61^ ^]^ for 2 micron‐sized 5 nm Au/(60 nm Ni80Fe20)/5 nm Au disks (80 % viability for 50 particles administered per glioma cell), indicating that ion leaching from non‐encapsulated MDPs might be a more wide‐spread phenomenon, or that parts of the cytotoxicity are caused by the rigid disk shape of the particles. To evaluate the latter, we synthesized disk shaped AlZr particles with a very similar morphology compared to the SAF MDPs. Without cobalt and samarium the disks with similar volume and shape had a density approximately half of that of their magnetic counterpart. Despite the lower density, at the same mass concentration the AlZr disks were substantially less toxic than the SAF MDPs with and without coating. In fact, the viability of macrophages with 24 h incubation with 10μg/mL AlZr disks was still around 93 ± 2 %. Therefore, we concluded that not the rigid disk shape is causing the cytoxicity towards macrophaegs, but rather the intracellular leaching of Co and Sm. This effect can be counteracted by a silica coating as we have demonstrated. Note that a significant effect of Si‐encapsulated MDPs on metabolic activity becomes evident for administered dose of 5μg/mL or higher, corresponding to 130 MDPs per cell (Figure 8c). This effect on cell viability is noticable, especially compared to typically used iron oxide based nanoparticles where doses exceeding 500μg/mL are typically well tolerated. Hence, for in vivo applications, further investigations into the origin of this effect are imperative.

### 2.7 High Performance Magnetic Separation by SAF MDPs

Finally, and after confirming the stability and cytocompatibility of the SAF MDPs, we performed magnetic separation experiments under flow conditions. We performed benchmarking experiments using the gold standard magnetic iron oxide beads (Adembeads with 160 nm TEM diameter and 200 nm hydrodynamic diameter) widely used for magnetic capturing in extracorporeal magnetic blood purification (Kang et al.^[^ ^50^ ^]^) and compared the magnetic separation efficiencies to our SAF MDPs at a flow rate of 1 mL/min, comparable to previous studies.^[^ ^50^, ^62^ ^]^ Capturing effiencies for Adembeads were just below 25% while the SAF MDPs where recovered at over 93% efficiency using simple permanent magnetic placed next to tube. When an optimized magnetic separator based on a Halbach design was employed, a quantitative separation of 99 % was obtained for SAF MDP at conditions whereas only around 60 % of the Adembeads were recovered (Figure 9). These experiments indicate clear superiority in terms of particle recovery by magnetic separation under flow conditions. In contrast to earlier work^[^ ^63^ ^]^ based on Fe/Ti/Ag SAF MDPs for water purification, our system design features a significantly higher ratio of magnetic‐to‐non‐magnetic material, translating to important performance benefits (e.g., quantitative capturing under flow instead of 5 mins in batch mode). The flow rates employed here are compatible and necessary for high‐throughput operations, e.g., during diagnostic or therapeutic target isolation. The efficiency of magnetic separation increases with a high ratio of magnetic to viscous forces acting on the particles in an applied magnetic field and field gradient. Since the viscous forces for both spherical and disk‐shaped particles scale linearly with the sphere or disk radius r, while the magnetic moment scales as $r^{3}$ for spheres and as $r^{2}\cdot t$ for disks (where t is the disk thickness), larger particle or disk dimensions are advantageous. However, for superparamagnetic particles, the particle radius must remain below approximately 15 nm to retain superparamagnetic properties. To overcome this limitation, multiple small superparamagnetic particles can be incorporated into a larger spherical carrier particle (e.g., realized by Adembeads), thereby increasing the total magnetic moment while preserving superparamagnetism. In contrast, SAF‐MDP structures can be designed in (almost) arbitrary sizes and volumes while maintaining a net‐zero magnetic moment in the absence of an external field. In addition the metallic ferromagnetic layers of SAF‐MDP exhibit a much larger magnetic moment (even in small applied fields) that the saturation moment of iron oxide particles contained in Adembeads. Moreover, SAF‐MDP can be designed to switch from their antiferromagnetically aligned into a high moment ferromagnetically aligned state in a small field (in our work about 40 mT), while the M(H) response of superparamagnetic particles follow a Langevin function reaching only a fraction of their saturation moment in a reasonably large magnetic field (< 1 T) that can be easily obtained with a permanent magnetic assembly. Furthermore, the volume fraction of magnetic material in Adembeads is lower than that of a compact metallic magnetic sheet embedded within a disk‐shaped particle, further enhancing the performance of SAF‐MDP.

## 3 Conclusion

In this work, we demonstrated the micromagnetic modelling‐assisted design and scalable manufacturing of synthetic antiferromagnet disk particles for high‐performance magnetic separation in comparison to widely used metal oxide beads. We demonstrate that such particles indeed unify the high colloidal stability needed for application with efficient, quantitative recovery even under flow, and thus overcome a main drawback of existing metal oxide‐based beads. In addition to promising performance benefits, we also uncover unexpectedly high effects on cell viability of such disk particles in line with previous work,^[^ ^61^ ^]^ even after silica encapsulation. This has important implications for the entire field of (magnetic) disk particles in biomedical applications, prompting a detailed evaluation of the risk/benefit ratios of such designer particles for in vivo use. Nonetheless, this work demonstrates convincing performance of SAF MDPs and quantitative capturing under flow conditions, an important prerequisite for high‐throughput applications in (in vitro) diagnostics and beyond.

## 4 Experimental Section

### Materials

Multi‐layer Stack: AlZr, Sm, and Co sputtering targets, all with a purity of 99.99 %, from HMW Hauner were used for the sputter deposition of the layers. Polysterene bead mask: Polystyrene Carboxylate‐Modified Nanospheres were purchased at Polyscience and used as a ion‐milling etching mask. Cells: The THP‐1 cell line (TIB‐202) was purchased from American Type Culture Collection. Surface Functionalization: Ammonia (NH4OH) solution 25 % was purchased from Carl Roth, ethanol ACS Reagent ≥99.8 %, was purchased from Honeywell and both tetraethyl orthosilicate (TEOS), ≥99.0 % and (3‐Aminopropyl)triethoxysilane (APTES), ≥98.0 % were purchased from Sigma–Aldrich.

### Micromagnetic Modelling

The micromagnetic simulations were carried out with the python library magnum.np [1], which solves the magnetization dynamics with the finite‐difference method by integrating the Landau‐Lifshitz‐Gilbert equation. A reasonably fine mesh‐size was chosen to approximate the disk shape of the particle. The dimensions of the disk in the simulations were 500×500×14 nm and discretized with a rectangular mesh of 250×250×7 cells, resulting in a cell size of 2×2×2 nm^3^. The effective field acting on the magnetic moments was composed of the exchange field, the anisotropy field, resulting from the uniaxial crystalline anisotropy, and the demagnetization field. The demagnetization field was responsible for the coupling of the magnetic layers, as well as for the formation of inhomogeneous states. Additionally, an external field was applied, which changes at a rate of one milli‐Tesla per nano‐second up to 100 mT. The field then cycles 1.5 times between 100 mT and ‐100 mT in order to create the hysteresis loops. In the non‐magnetic spacer layer the exchange constant, anisotropy constant, and magnetization were set to zero, to ensure that the layers were not exchange coupled. The stochastic temperature effects were omitted in these micromagnetic simulations. Temperature would lead to reversal via a finite energy barrier that would lower the coercive field. In order to break the symmetry, which could get the time‐integration initially stuck on the maximum in the energy landscape, the external field was applied at an angle of $1^{\circ }$. Typical micromagnetic modeling usually did not include temperature effects, which significantly influence the magnetic behavior of superparamagnetic nanoparticles. In these nanoparticles, thermal energy at room temperature easily overcomes the small anisotropy energy barriers, resulting in superparamagnetic behavior. When cooled, these particles exhibit hysteresis and remanence, similar to ferromagnets, due to the reduced thermal activation. However, in larger SAF magnetic disk particles (SAF MDP), the anisotropy energy barrier was high enough at room temperature to maintain an antiferromagnetic ground state unless switched by strong external fields, with thermal energy only minimally lowering the switching field. Factors like domain wall pinning and slight size variations play a more significant role in the switching behavior, explaining minor discrepancies between micromagnetic models and experimental observations (VSM). Micromagnetic modeling was therefore primarily employed to narrow down critical parameters before experimental exploration.

### Fabrication of SAF MDP

SAF MDPs were fabricated by sputter‐deposition and microfabrication using 2‐inch Si wafers as a substrates. All fabrication steps are illustrated in Figure 3a–f. As a sacrificial layer a 50 nm Ge layer was evaporated on top of a 2‐inch silicon wafer. The multi‐layers including the top sacrificial layer of 7 nm MgO were then grown on a 2‐inch Si wafer using a AJA DC/RF magnetron sputtering system with a working pressure of ≈ 2 μbar Ar and a base pressure of $\approx \;1\times 10^{-8}\mathrm{mbar}$. To induce a well defined uniaxial in‐plane magnetic anisotropy, the substrate was mounted on an in‐house build sample holder containing two NdFe permanent magnets producing a uniform field of 40mT in the plane of the substrate. On top of the MgO layer a hard mask out of carboxylate‐modified polysterene (PS) beads with a diameter of 660 nm was self‐assembled at the water‐air interface. This method^[^ ^32^, ^49^ ^]^ created a highly ordered hexagonal packed monolayer of PS beads which was deposited on top of the multi layer stack by slow water evaporation. In a next step the PS beads were treated with oxygen plasma for 8min 30s with a power of 40W to separate them out and to reach the diameter of 500 nm. To cut the disk out of the multi layer stack an Ar ion‐miller from Oxford Instrument was used with a working pressure of ≈ 3.5 × $10^{-3}\mathrm{mbar}$. The milling was performed at 600 V with 300 mA at an angle of $90^{\circ }$ to the sample surface. The MgO top sacrificial layer was then removed after 30 min in citric acid and the Ge bottom sacrificial layer was dissolved in a final step during 1.5 h in 35wt.% $H_{2}O_{2}$ to release the SAF MDP. Moreover, stacking of the multi layers with intermediate sacrifical MgO layer could double the amount of MDPs per wafer. After dissolving the MDPs from the wafer the MgO could be etched again with citric acid and ultrasonication for more than 30 min.

### Demagnetization of SAF MDP

To reach stable suspensions, the SAF MDP were demagnetized. In order to block the disks physically, 2 mL a SAF MDP suspension in pure water was frozen in liquid nitrogen at ${-196}^{\circ }C$ for approximately 3 min. The frozen suspension was kept in an externally applied, alternating decreasing magnetic field with a initial field amplitude of 50 mT, a frequency of 5 Hz and a decaying time of 30 s.

### Quantification of Separation Efficiency Under Magnetic Field

To assess the separation efficiency under a magnetic field, two demagnetized 1 mL suspensions were prepared, each containing approximately 70 μg of magnetic material, one of SAF MDPs and the other of Adembeads. These suspensions underwent ultrasonication for 10 min. Subsequently, the samples were filtered using a Hallbach array magnet arrangement consisting of NdFeB cube magnets (Supermagnete, 1.37 T) for 1 min at a flow rate of 1 mL/min. The filtered materials were then collected in separate Eppendorf tubes. The content of Co, Sm, Al, and Zr in each sample was quantified using Inductively Coupled Plasma Mass Spectroscopy (ELEMENT 2 ICP‐MS, Thermo Scientific) to determine the separation efficiency.

### Scanning Electron Microscopy (SEM) and Scanning Transmission Electron Microscopy (STEM)

SEM was performed on the SAF MDPs immobilized on the wafer and following detachment using a Hitachi SU5000 with a tilt stage. Detached SAF MDPs were subsequently transferred onto carbon coated copper grids for observation by STEM performed at Scope M, ETH Zurich. The samples were examined using a FEI Talos F200X operating at 200 kV and equipped with a Super‐X energy dispersive X‐ray spectroscopy (EDXS) detector (four‐detector configuration, Thermo fisher scientific). Elemental maps were processed and evaluated using the software Velox (Version 3.0.0.815, Thermo fisher scientific) Particle size distributions were analyzed based on TEM micrographs (>200 particles were manually counted per sample).

### Atomic Force Microscopy

Atomic force microscopy (AFM) of the MDP attached to the wafer was performed in tapping mode using a Bruker Dimension Icon three with RTESPA‐300 probes (Bruker). The images were acquired at a scan rate of 0.5 Hz and a resolution of 1024x1024 lines. The raw data was flattened with the Bruker Nanoscope Analysis software, which was also used to extract height profiles of the MDPs.

### Magnetometry Measurements

The bulk magnetic properties, including the magnetization were determined by a 7 T “Quantum Design” vibrating sample magnetometer (VSM). The in‐plane measurements were performed at 300K up to a field of 3 T. Furthermore the easy‐axis in‐plane magnetization loops were measured with a Kerr Microscopy (MOKE) from “evico magnetics”.

### Time‐Of‐Flight Secondary Ion Mass Spectrometry (ToF‐SIMS) Measurement

For compositional analysis, secondary ion mass spectrometry measurements were conducted on a ToF‐SIMS.5 from IONTOF GmbH, Germany. The instrument was operated in spectroscopy mode, and ${\mathrm{Bi}}^{3+}$ primary ions with an energy of 25 keV were used to analyze areas of 50 × 50 $\;\mu m^{2}$, for the construction of images and for depth profiling. Depth profiles were measured by sputtering the surface with Cs with an energy of 1 keV. An area of 500 × 500 $\;\mu m^{2}$, was sputtered while the positive ions on an area of 50×50 $\;\mu m^{2}$, were detected.

### SAF MDP Elemental Analysis and Chemical Stability

The total elemental composition, as well as the chemical stability (i.e., dissolution) of the MDPs in relevant media was additionally determined using inductively coupled plasma mass spectrometry (ICP‐MS). In a first step, the MDPs were detached from the wafer using H_2_O_2_ and washed at least three times with Milli‐Q water. To determine the total element concentrations, from such a sample, 25–100μL (depending on the available sample volume) were digested in quartz tubes using a mixture of 0.75 mL hydrochloric acid (37 pc, Normatom, VWR) and 0.25 mL nitric acid (69 pc, Normatom, VWR) in a pressurized microwave (TurboWAVE, MLS GmbH, Germany) at 230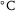 and 120 bar for 19 min. Subsequently, the samples were filled into 50 mL Falcon tubes and filled up with ultrapure water to the mark. All samples were analyzed for Mg, Al, Co, Zr, and Sm using a 7900 single‐quadrupole ICP‐MS (Agilent Technologies, CA). The isotopes 24Mg and 27Al were measured in No‐gas mode, whereas 59Co, 90Zr and 147Sm were determined in He‐collision mode. Non‐spectral interferences were corrected using an internal standard, containing Li (to correct Mg and Al) and Rh (for all other elements), which was mixed online with the sample. Calibration was performed with certified element standards (Inorganic ventures) diluted in the same acid matrix as the samples (0.75 mL HCl/0.25 mL HNO_3_ in 50 mL ultrapure water). Samples containing cells were analyzed using the same methodology. Dissolved fractions were operationally defined as material able to pass through a 10 kDa filter. To determine dissolved fractions, samples were split into two. One part was used for total element content determination as described above and the other part was filtered through a 10 kDa PES centrifugal filter. The filtrate was subsequently diluted in the used acid matrix (see above) and its elemental content determined using ICP‐MS.

### Silica Encapsulation and Surface Functionalization

SAF MDP were enveloped with amorphous silicium dioxide using an adapted Stöber sol–gel process according to Kobayashi, et al.,^[^ ^64^ ^]^ Therefore, SAF MDPs of around 60μg of total particle mass, were dispersed under ultrasonic conditions in a total volume of 800μL Milli‐Q water. The particle solution was injected in 14.28mL ethanol and sonicated for 15 min. Subsequently, the pH was adjusted by adding 80μl NH_4_OH solution, the mixture shortly vortexed, and sonicated for 15 min to ensure a homogenous solution. Afterwards, a total amount of 200μL TEOS, divided into two 100μL injections, was added to the suspension.

The injections were separated by a 12h break, and the reaction was not finished before 6h after the last addition to guarantee well‐dispersed and uniformly coated particles. After each addition, the mixture was sonicated for 15min and shaken (Unimax 2010, 320rpm) to prevent attraction to a magnetic stirring bar. The reaction was terminated by removing the SAF MDPs from the initial reaction solution using a strong magnet. The particles were purified by discarding the supernatant and washing with ethanol (3 times, 5mL each) and water (3 times, 5mL each). Finally, the particles were dispersed in a total amount of 1200μl Milli‐Q water.

The silica‐coated particles provided a versatile platform and were further modified by introducing APTES as an amine‐containing linker. Due to structural similarities, the previously described protocol was used with minor alterations. Both TEOS injections were substituted by a single injection of 75μl APTES, followed by 15 min sonication, and the reaction was shaken overnight (Unimax 2010, 320rpm). After this, the reaction was terminated, and the particles were purified as described before.

### X‐Ray Photoelectron Spectroscopy

X‐ray photoelectron spectroscopy (XPS) data was acquired using a Physical Electronics (PHI) Quantera SXM (ULVAC‐PHI, USA) equipped with a hemispherical capacitor electron‐energy analyzer. All the reported analyses were performed by a monochromatic source of Al Kα(1486.6eV), a nominal spot size of 200μm for the X‐ray beam, and $45^{\circ }$ emission angle in constant‐analyzer‐energy (CAE) mode under a low‐voltage electron neutralizer/argon‐ion gun with low voltage active. Calibration of the spectrometer was performed according to ISO 15472 (2010) for the binding energies of Cu 2p3/2, Ag 3d5/2, and Au 4f7/2 observed on ion‐etched pure copper, silver, and gold, respectively. The presented spectra were analyzed with CasaXPS, where a Shirley estimation was used to subtract the background from the peak area. The survey and high resolution spectra were acquired with 280 eV (1 eV step size) and 55 eV (0.05 eV step size) pass energies, respectively. Curve fitting of the high resolution spectra was carried out using a product function of Gaussian/30 % Lorentzian for building the synthetic line shapes.

### Particle Cytotoxicity and Cellular Uptake

For cytotoxicity and uptake studies of SAF MDP, THP‐1 cells were seeded in 96 well plates ($4x10^{4}$ cells per well) or six well plates ($1x10^{6}$ cells per well) and treated with 200 nM PMA for 72h to differentiate the human monocytes into adherent macrophages. The PMA was removed and cells were washed with fresh cell culture medium prior to adding the SAF MDPs suspended in cell culture medium at different concentrations ranging from 0.01 to 10μg/mL. The SAF MDPs were incubated with the cells in a standard cell culture incubator for 24 h. Triton X (1 %) served as a positive cell‐lysis control. After 24 h, the cell viability was analyzed using the CellTiterGlo luminescence assay (Promega) according to the manufacturer's protocol.

### Cell Sample Preparation for TEM Imaging

For electron microscopy, cells were cultured as above and chemically fixed with 4 % methanol‐free paraformaldehyde containing 0.25 % of glutaraldehyde in PBS for 24 h. Afterwards, cells were washed with cacodylic buffer and stained with 2 % osmium tetraoxide for 2 h. Following the OsO_4_ removal, cells were dehydrated using an ethanol gradient and embeded in epoxy resin (EPON, Simga). The resin was cured at 60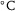 for 48h and resin blocks were sectioned into 100 nm sections using an ultramicrotome and transferred to copper grids. Thin sections were imaged on a JEOL 120kV TEM, as well as a FEI TALOS F200X for EDXS mapping.

## Conflict of Interest

The authors declare no conflict of interest.

## Supporting information

Supporting Information

## Data Availability

The data that support the findings of this study are available from the corresponding author upon reasonable request.
