# Supplementary material for: Synthetic Antiferromagnetic Designer Nanodisks for High‐Performance Magnetic Separation
Source: Adv Healthc Mater. 2025 Aug 11;14(31):2500616. doi: 10.1002/adhm.202500616 (PMC12683210; doi:10.1002/adhm.202500616)
Supplement: Supplementary file 1 — Supporting Information [file ADHM-14-0-s001.pdf]

ADVANCED HEALTHCARE MATERIALS

Supporting Information

for *Adv. Healthcare Mater.*, DOI 10.1002/adhm.202500616

Synthetic Antiferromagnetic Designer Nanodisks for High-Performance Magnetic Separation

Subas Scheibler, Sebastian Habermann, Alexander Gogos, Santiago Helbig, Lukas R. H. Gerken, Anna L. Neuer, Vera M. Kissling, Michal Krupinski, Mohammad Alinezhadfar, Rowena Crockett, Nico Kummer, Erik M. Mayr, Dieter Süss, Hans J. Hug and Inge K. Herrmann**

Synthetic Antiferromagnetic Designer Nanodiscs for High-Performance Magnetic Separation

S. Scheibler[a,b,c], S. Habermann[a,b,d,e], A. Gogos[a,b,d,e], S. Helbig[f,g], L. R. H. Gerken[a,b,d,e], A. L. Neuer[a,b,d,e], V. Kissling[b], M. Krupinski[h], M. Alinezhadfar[i,j], R. Crockett[i], N. Kummer[k,1], E. Mayr[a,b,c,d,e], D. Süss[f,g], H. J. Hug[*,c,m], I. K. Herrmann[*,a,b,d,e]

[a] *Nanoparticle Systems Engineering Laboratory, Institute of Energy and Process Engineering (IEPE), Department of Mechanical and Process Engineering (D-MAVT), ETH Zurich, Sonneggstrasse 3, 8092 Zurich, Switzerland*
[b] *Nanomaterials in Health Laboratory, Department of Materials Meet Life, Swiss Federal Laboratories for Materials Science and Technology (Empa), Lerchenfeldstrasse 5, 9014 St. Gallen, Switzerland.*
[c] *Magnetic & Functional Thin Films Laboratory, Department of Materials Meet Life, Swiss Federal Laboratories for Materials Science and Technology (Empa), CH-8600 Dübendorf, Switzerland*
[d] *Faculty of Medicine, University of Zurich, Raemistrasse 71, 8006 Zurich, Switzerland*
[e] *Ingenuity Lab, Balgrist University Hospital, Forchstrasse 340, 8008 Zurich, Switzerland*
[f] *Physics of Functional Materials, Faculty of Physics, University of Vienna, Vienna, Austria*
[g] *Research Platform MMM Mathematics – Magnetism – Materials, University of Vienna, Vienna, Austria*
[h] *Institute of Nuclear Physics Polish Academy of Sciences,Department of Magnetic Materials and Nanostructures, Krakow, Poland*
[i] *Surface Science and Coating Technologies Laboratory, Department of Advanced Materials and Surfaces, Swiss Federal Laboratories for Materials Science and Technology (Empa), CH-8600 Dübendorf, Switzerland*
[j] *Durability of Engineering Materials, Institute for Building Materials (IfB), Department of Civil, Environmental and Geomatic Engineering (D-BAUG), ETH Zurich, 8093 Zurich, Switzerland*
[k] *Laboratory of Food Process Engineering, Institute of Food, Nutrition and Health (IFNH), Department for Health Sciences and Technology (D-HEST), ETH Zurich Schmelzbergstrasse 9, 8092 Zurich, Switzerland*
[l] *Laboratory for Cellulose and Wood Materials, Department of Materials Meet Life, Swiss Federal Laboratories for Materials Science and Technology (Empa), Überlandstrasse 129, 8600 Dübendorf, Switzerland*
[m] *Department of Physics, University of Basel, CH-4056 Basel, Switzerland*

[*] Corresponding authors: hans-josef.hug@empa.ch and ingeh@ethz.ch

Supplementary Information

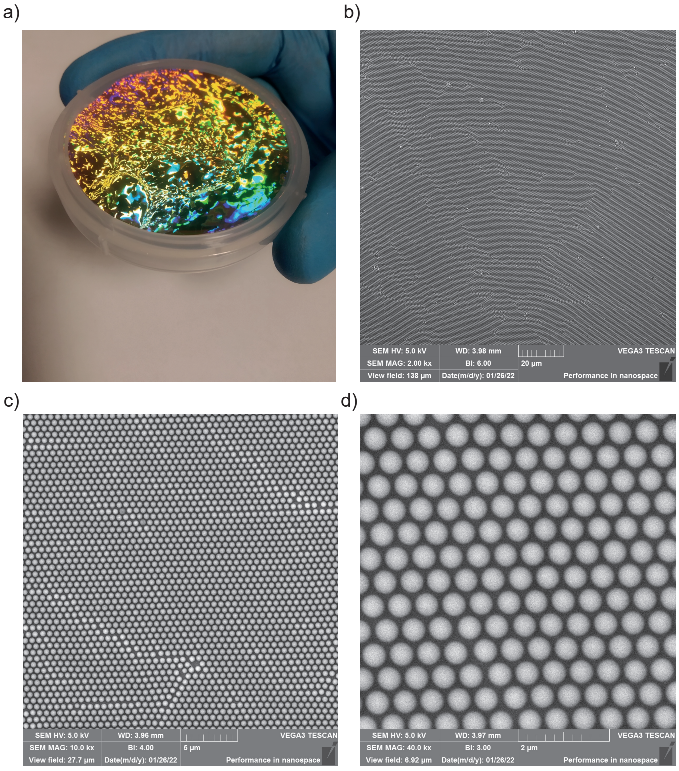

Figure S1: a) 2-inch wafer featuring multilayers covered by a hexagonal close-packed monolayer of PS beads. b) to d) SEM images of the PS bead ion milling mask after reducing their diameter to 500 nm, displaying high order and minimal defects.

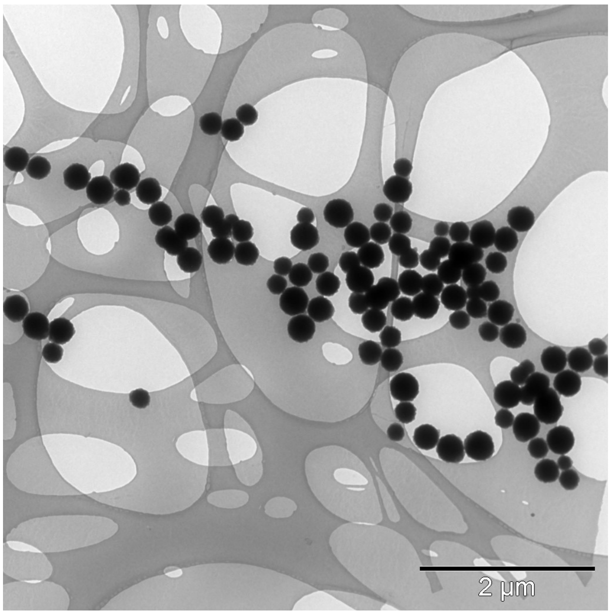

Figure S2: Transmission electron micrograph of Adembeads
